# New Insights Into Potential Benefits of Bioactive Compounds of Bee Products on COVID-19: A Review and Assessment of Recent Research

**DOI:** 10.3389/fmolb.2020.618318

**Published:** 2021-02-08

**Authors:** Ehab Kotb Elmahallawy, Yasser Mohamed, Walied Abdo, Fatma A. El-Gohary, Shaimaa Ahmed Awad Ali, Tokuma Yanai

**Affiliations:** ^1^ Department of Zoonoses, Faculty of Veterinary Medicine, Sohag University, Sohag, Egypt; ^2^ Laboratory of Kafr El Sheikh Fever Hospital, Kafr El Sheikh Fever Hospital, Kafr El-Sheikh, Egypt; ^3^ Department of Pathology, Faculty of Veterinary Medicine, Kafrelsheikh University, Kafr El-Sheikh, Egypt; ^4^ Department of Hygiene and Zoonoses, Faculty of Veterinary Medicine, Mansoura University, Mansoura, Egypt; ^5^ Department of Nursing, College of Applied Medical Sciences, Jouf University, Sakaka, Saudi Arabia; ^6^ Department of Critical Care and Emergency Nursing, Faculty of Nursing, Mansoura University, Mansoura, Egypt; ^7^ Laboratory of Wildlife and Forensic Pathology/Biomedical Science Examination and Research Center, Department of Veterinary Medicine, Faculty of Veterinary Medicine, Okayama University of Science, Okayama, Japan

**Keywords:** COVID‐19, pathogenesis, honey bee, products, inhibitors

## Abstract

The recent emergence of COVID‐19 represents one of the biggest challenges facing the world today. Despite the recent attempts to understand the epidemiological pattern and pathogenesis of the disease, detailed data about the physiology and pathology of the disease is still out of reach. Moreover, the lack of a widespread vaccine prompts an urgent call for developing a proper intervention strategy against the virus. Importantly, identification of novel molecules that target replication of the virus represents one of the promising strategies for the control this pandemic crisis. Among others, honey bee products contain numerous bioactive compounds such as propolis and several phenolic compounds that possess a wide range of therapeutic properties for combating various pathological disorders and infectious agents. The intention of the present review is to highlight the stages of SARS-CoV-2 lifecycle, the molecular mechanisms explaining the health benefits of honey bee products on COVID‐19 physiology and pathology and the possible limitations. Further future research is suggested to explore more about bee natural bioactive compounds as potential candidates against SARS-CoV-2.

## Introduction

Corona viruses (CoV) are a group of positive-sense single-stranded RNA viruses ranging between 26 and 32 kb in size (Graham et al., 2013; Song et al., 2019). This group of viruses belongs to genus β-Coronavirus, family Coronaviridae, and order Nidovirales (Paules et al., 2020). The epidemiological profile of Corona viruses’ infection involves a wide range of hosts that include humans, birds, and other mammals (Monchatre-Leroy et al., 2017; Cui et al., 2019). The clinical impact of these viruses ranges from asymptomatic cases to severe symptoms, affecting respiratory, digestive, and genital organs. In accordance with COVID‐19, the disease has been emerged and detected for the first time in patients with respiratory illness of unknown etiology in the urban center town, Hubei Province, Central China (Graham et al., 2013; Song et al., 2019; Paraskevis et al., 2020). The viral agent was defined as coronavirus illness 2019 (COVID-19) or SARS-CoV-2 (Li et al., 2020b; Sun et al., 2020; Xu et al., 2020). As of December 15, 2020, more than 73,212,302 confirmed cases of COVID-19 and 1,628,442 deaths have been reported worldwide (Worldometer, 2020). Interestingly, honeybee products have been used in treatment of many diseases including tumor and immune-related diseases (Yusuf et al., 2007; Wieckiewicz et al., 2013). In this regard, honey, propolis, Bee pollen and Bee venom created by bees possess many biological activities like antibiotic, antifungal, antioxidant, antiviral, inhibitor, anti-cancer, and immunomodulatory, hepatoprotective effects (Banskota et al., 2001; Tolba et al., 2013; Pasupuleti et al., 2017; Hashem, 2020; Shaldam et al., 2020). The following sections include an overview about structure, pathogenesis and mechanistic activities of SARS-CoV-2 and the potential application of Bee’s products in treatment of the disease.

## Structure of SARS-CoV-2

**FIGURE 1:**
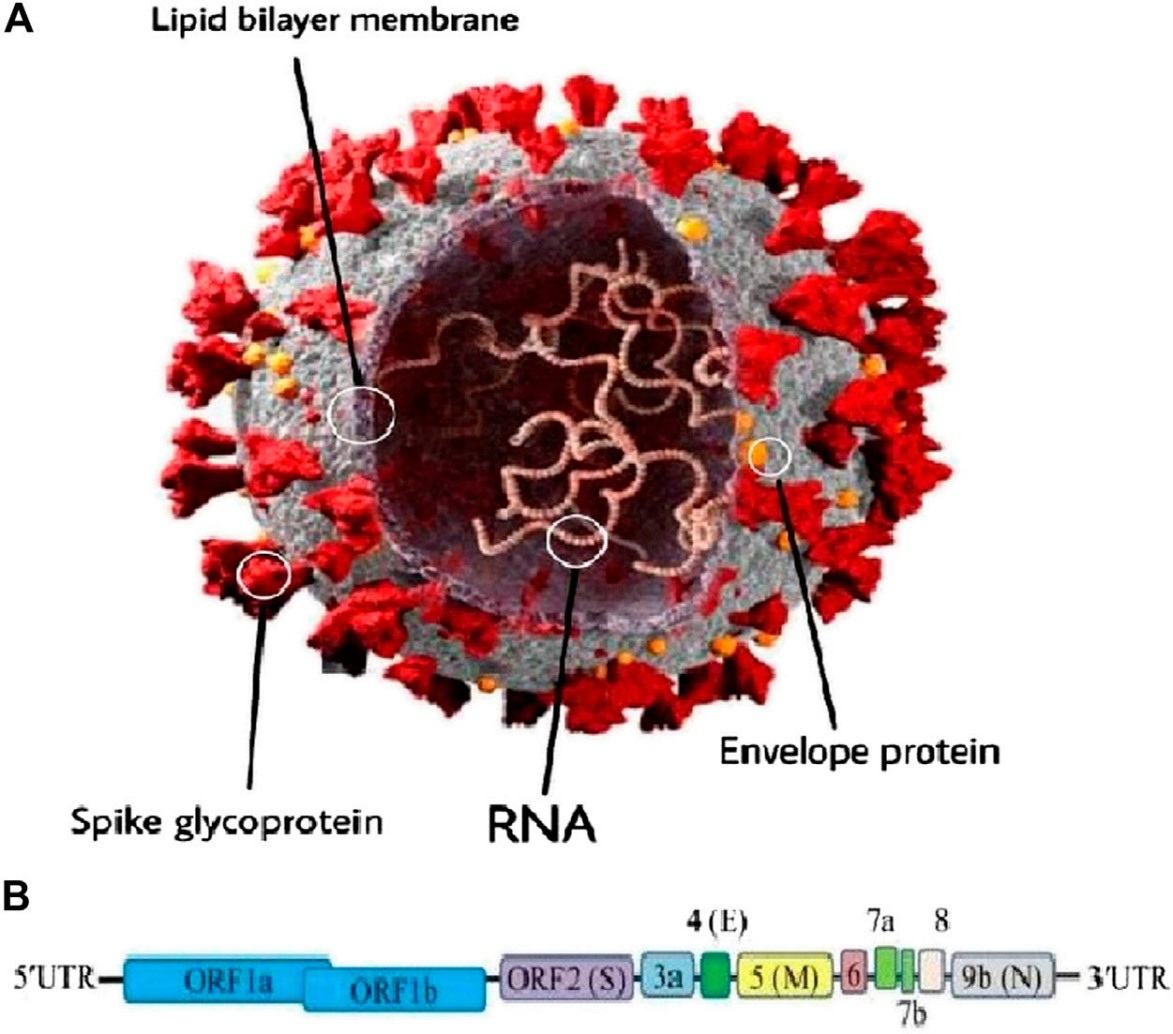
**(A)** Schematic representation of the genome organization and functional domains of S protein for COVID-19. The single-stranded RNA genomes of COVID-19 encode two large genes, the ORF1a and ORF1b genes, which encode 16 non-structural proteins (nsp1–nsp16). The structural genes encode the structural proteins, spike (S), envelope **(E)**, membrane (M), and nucleocapsid (N), which are common features to all coronaviruses. **(B)** The SARS-CoV-2 genome is arranged in the order of 5′-replicase (ORF1a/b)–structural proteins [spike (S)–envelope **(E)**–membrane (M)–nucleocapsid (N)]−3′ (reproduced from Consortium, 2004; Peiris et al., 2004; Li et al., 2020a; Nadeem et al., 2020; Wrapp et al., 2020).

As mentioned above, SARS-CoV-2 or CoV is an RNA virus belonging to the genus β-Coronavirus (Paules et al., 2020). This virus is a positive-sense RNA virus with a size of around 30 kb and about 74–99% identity with the coronavirus from the placental mammal (Manis javanica) and horseshoe bat (Rhinolophus sinicus) (Bat-CoVRaTG13), respectively (Zhu et al., 2020b). The typical CoV contains a minimum of six open reading frames (ORFs) (Consortium, 2004). The primary ORF (ORF1a/b) is a simple fraction that concerns the entire order length and encodes sixteen non-structural proteins (nsp1-16) (Consortium, 2004; Narayanan et al., 2015). Furthermore, the virus includes four main structural proteins; spike (S), envelope (E), membrane (M), and nucleocapsid (N) proteins which are shown in Figure 1 (Consortium, 2004; Peiris et al., 2004; Li et al., 2020a; Nadeem et al., 2020; Wrapp et al., 2020). It should be stressed that most of the non-structural proteins are known to play a significant role in virus replication while structural proteins are vital for particle assembly and for inflicting CoV infection (Consortium, 2004; McBride et al., 2014; Astuti and Ysrafil, 2020). Moreover, specific structural and accent proteins, like HE macromolecules are also encoded by CoV (Chen et al., 2020a; Naqvi et al., 2020).

## Stages of SARS-CoV-2 Lifecycle and the Potential Inhibition Targets

**FIGURE 2:**
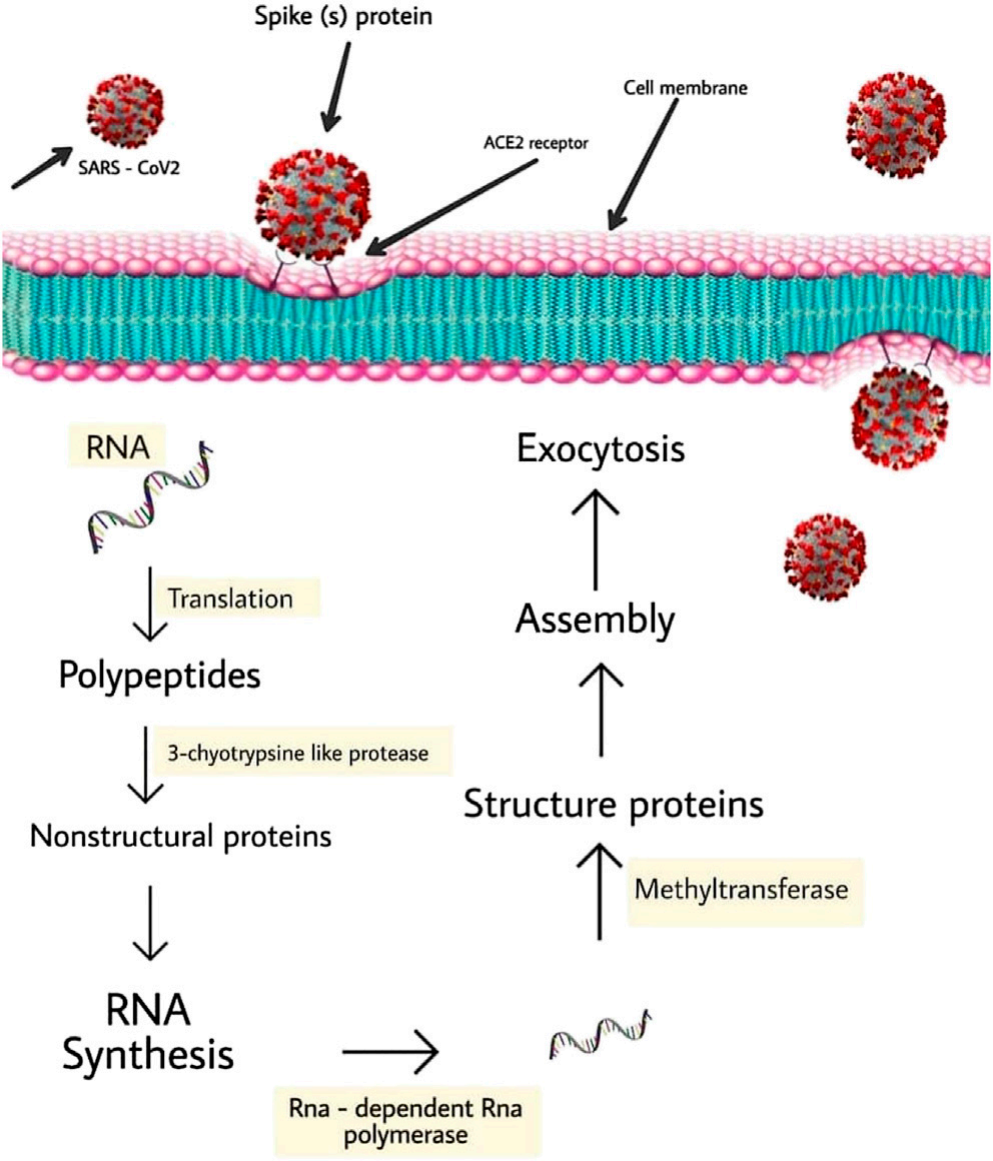
Schematic represents virus-induced host immune system response and viral processing within target cells.

It is noteworthy to state that SARS-CoV-2 targets cells through the infectious agent structural spike (S) supermolecule that binds to the angiotensin-converting enzyme two (ACE2) receptor (Wang et al., 2007; Magrone et al., 2020). Following its binding to the receptor, the virus uses host cell receptors and endosomes to enter cells while this action is facilitated by transmembrane protease/serine subfamily member 2 via the S supermolecule (Hoffmann et al., 2020b). Once within the cell, the infectious agent polyproteins area unit synthesized targets the assembly of replicase-transcriptase complex (Sawicki et al., 2005). The virus then synthesizes RNA via its RNA-dependent RNA enzyme and the structural proteins area unit synthesized, resulting in completion of assembly which is followed by the release of infectious agent particles (Fung and Liu, 2014; Fehr and Perlman, 2015; Chen et al., 2020a). These steps of the SARS-CoV-2 lifecycle represent potential drug targets (Figure 2) and there are other drug targets which trigger infectious agent entry and immune regulation pathways (Savarino et al., 2003; Al-Bari, 2017).

## The Role of Natural Therapy Strategy in SARS-CoV-2

Revising the available literature, several previous studies explored the promising role of some natural compounds and phytochemical extracts, e.g. Lycoris radiata (red spider lily), Lindera aggregata, Pyrrosia lingua (a fern), and Artemisia annua (sweet wormwood), in treatment of outbreaks of SARS (Wu et al., 2004; Hoever et al., 2005; Li et al., 2005; Kim et al., 2010). Taken into account, these previously mentioned extracts showed various degrees of activity against SARS-CoV ranged from moderate to potent and their antiviral actions were dose-dependent. Among others, Lycoris radiata (red spider lily) expressed the most potent antiviral activity (Li et al., 2005). Glycyrrhizin, which is an active compound contained in licorice roots, is another example for the herbal extracts that displayed potent antiviral activity against SARS-CoV by inhibiting the replication of the virus when tested on 10 different clinical strains of SARS-CoV (Cinatl et al., 2003; Hoever et al., 2005). Furthermore, Lycorine, a toxic crystalline alkaloid found in various Amaryllidaceae species and Baicalin (a constituent of the Baikal skullcap plant), has also shown potent antiviral effects against SARS-CoV (Fielding et al., 2020). Interestingly, myricetin, scutellarein, and phenolic compounds from dyer’s woad and Japanese nutmeg-yew have shown to be potent antagonists of SARS-CoV enzymes, including nsP13 helicase and 3CL protease (Lin et al., 2005; Ryu et al., 2010; Yu et al., 2012). In addition, some natural phytomedicines, e.g., the aqueous extract of fish mint, mediated several antiviral mechanisms in SARS-CoV (Lau et al., 2008). However, it should be borne in mind that some discrepancy in results has been reported as these *in vitro* data may not correlate with *in vivo* findings that renders the use of some of these natural compounds as an effective antiviral agent. The following subsections will highlight the potential inhibition targets in the different stages of SARS-CoV-2 lifecycle and the potential application of bee products in treatment of the disease.

### Potential Repressive Properties Against ACE-2 Receptors

The intra- or inter-species transmission of β-coronaviruses (CoVs) requires an interaction between the infective agent and the host cell receptors that results in the invasion of the virus into host cells (Li, 2016). Some recent studies reveal that human, pig, and civet cell lines allowed SARS-CoV-2 infection and replication, indicating that the virus uses ACE2 receptor for infection (Hoffmann et al., 2020a; Zhu et al., 2020a; Zhu et al., 2020b; Letko et al., 2020; Zhou et al., 2020). ACE2 is extremely expressed within the respiratory organs that make the lung tissue highly vulnerable (Hamming et al., 2004). In addition, the ACE2 receptor is expressed within the epithelial tissue cells of gut, kidney, and heart cells (Zhang and Liu, 2020). It is therefore not surprising to state that ACE2 blockers are another choice to control the infection (Ton et al., 2020). Similarly, some molecules such as GSK1838705A (a small-molecule kinase inhibitor), KT203 (inhibitor of α/β-hydrolase domain), KT185 (brain-penetrant and selective ABHD6 inhibitor), and BMS195614 (selective RARα antagonist) showed strong binding affinities with receptor binding sites (RBD) of the infective agent S-protein. These molecules facilitate the management of fast infection by participating the virus at entry points (Choudhary et al., 2020). Therefore, ACE II enzyme inhibition seems an important target for treatment of these cases of infection caused by SARS-CoV-2.

Propolis, or bee glue, is defined as a natural resinous mixture produced by bees through its collection from nature (Sforcin, 2016; Drescher et al., 2019). The honey bee produces this mixture though mixing saliva and beeswax together with the collected exudate from several botanical sources such as tree and plants buds (Zabaiou et al., 2017). Interestingly, this mixture possesses a wide range of activity against various infections agents in addition to its role in wound healing (Pasupuleti et al., 2017; Oryan et al., 2018). In accordance with its texture, crude propolis could be extremely viscous and slightly soluble in water. Propolis has been an important element of apitherapy for hundreds of years. Recently, it has been used as an additive in the name of the ancient practice of medicine (Pobiega et al., 2018; Anjum et al., 2019). The bulk of the active ingredients of propolis comprise the family of polyphenols. In this concern, phenolic acids, flavonoids (flavanones, flavones, flavonols etc.), stilbenes, and tannins are considered the most active polyphenols of propolis (Graikou et al., 2015; Anjum et al., 2019). In addition, several previous *in vitro* and *in vivo* studies showed that flavonoids have high potential for inhibition of Angiotensin-Converting enzyme (ACE) (Hussain et al., 2018; Wang et al., 2018; Silveira et al., 2019). A recent study measured and checked the binding constants of 10 flavonoids, including caffeic acid, caffeic acid phenethyl ester, galangin, chrysin, rutin, hesperetin, myricetin, pinocembrin, quercetin, and luteolin, using the AutoDock 4.2 molecular arrival program and compared to a reference substance of MLN-4760 which is known as ACE2 inhibitor (Güler et al., 2020). The results showed that rutin has the simplest inhibition potential among the studied molecules. Clearly, the high potential of flavonoids extracts to bind to ACE II receptors indicates that this natural bee product might exhibit marked activity for Covid- 19 treatment (Güler et al., 2020; Shaldam et al., 2020). However, these findings must be supported by experimental studies.

### Potential Repressing Properties Against Proteinase Enzyme

The inhibition of infectious agent proteinase is a crucial target in drug development. The 3C-like proteinase (3CLpro) might be a cysteine proteinase that hydrolyzes the polyproteins pp1a and pp1ab to supply purposeful proteins throughout the replication of the virus. As result of its extremely preserved sequence and essential properties, 3CLpro has been validated as a possible target for the treatment of respiratory illnesses such as MERS, and COVID-19 (Kumar et al., 2017; Dong et al., 2020; Jo et al., 2020). Recently, a wide range of natural and artificial inhibitors that focus on completely different sites and regions of 3CLpro have been developed (Muramatsu et al., 2016; He et al., 2020; Theerawatanasirikul et al., 2020; Ye et al., 2020). Because the extremely preserved process sites of 3CLpro area unit shared by CoVs (Kumar et al., 2017; Dong et al., 2020), tremendous efforts have been created to review this target in order to meet the urgent need for the development of anti-SARS-CoV-2 therapy (Ton et al., 2020). Their targets include antecedently approved medicine, run candidates, and bioactive agents that were known as potential treatments for respiratory illness and MERS (Ton et al., 2020). Most studies targeted the small-molecule compounds, through virtual screening, that supported the crystal structure of 3CLpro (Chen et al., 2020b). It should be stressed that the natural bee products such as flavonoids (herbacetin) and Chalcones are considered candidate compounds which are related to the protein activity or infectious agent load *in vitro*. In addition, Herbacetin (PubChem CID: 5280544) exerted outstanding repressing effects, with the IC_50_ values of 33.2 μM. An induced-fit docking tying up study with SARS-CoV 3CLpro (PDB ID: 4WY3) showed that herbacetin shaped four H-bonds at the S2 website besides the 8-hydroxyl cluster that was essential for the formation of H-bonds with Glu166 and Gln 189 (Jo et al., 2020).

Interestingly, several previous studies documented that galangin, kaempferol, chrysin, and pinocembrin were detected in Croatian Cystus incanus L. bee pollen (Saric et al., 2009). The presence of herbacetin, myricetin, tricetin, luteolin, and 3-O-methylquercetin was also documented (Campos et al., 2003). A series of alkylated chalcones isolated from Angelica keiskei, were evaluated for their repressing activities against SARS-CoV 3CLpro. Among these chalcones, compound, with a perhydroxyl cluster, showed the foremost potent repressing impact (IC_50_ = 11.4 ± 1.4 μM). Clearly, these previously mentioned results reveal that the perhydroxyl cluster can be crucial for the binding to SARS-CoV 3CLpro. Tying up studies of the compound with 3CLpro (PDB ID: 2ZU) showed that the carbonyl and hydroxyl group teams shaped H-bonds with His163 and Ser144, respectively. The perhydroxyl cluster shaped a powerful H-bond with the very important residue Cys145 (Park et al., 2016). It is noteworthy to state that honey contains various compounds besides water, sugars, free amino acids, proteins, enzymes, essential minerals, vitamins, and numerous phytochemicals (Escuredo et al., 2013). In addition, polyphenols are heterogeneous categories of chemical compounds which are divided into flavonoids (flavonols, flavones, flavanones, flavanols, chalcones, anthocyanidin, and isoflavones) and non-flavonoids (phenolic acids). The phenolic resin composition in honey mainly depends on its floral origin that can be used as a tool for classification and authentication, particularly within unifloral varieties (Kennedy and Wightman, 2011; Chan et al., 2013; Keckes et al., 2013; Campone et al., 2014). Importantly, two compounds of chalcones (2′, 4′- dihydroxychalcone and 2′,4′- dihydroxy 3′-methoxychalcone) were also found in propolis samples from the four-card monte phytogeographical region (Solórzano et al., 2012).

### Potential Restrictive Properties Against Methyltransferase

It should be stressed that cap formation of an mRNAs infectious agent requires universally three sequent accelerator reactions. Firstly, RNA triphosphatase (TPase) removes the c-phosphate cluster from the 59-triphosphate finish (pppN) of the emergent RNA chain to come up with the diphosphate 5ʹ-ppN. Later on, RNA guanylyltransferase (GTase) transfers a Guanosine monophosphate (GMP) to the 59-diphosphate to yield the cap core structure (GpppN). Then, N7- MTase methylates the capping guanylate at the N7 position to supply a cap-0 structure (m7GpppN) (Furuichi and Shatkin, 2000). Taken into account, lower eukaryotes such as yeast use a cap-0 structure while higher eukaryotes and viruses sometimes markedly methylate the cap-0 structure at ribose 2ʹ-O position of the first and second nucleotide of the mRNA via a ribose 2ʹ-O MTase, which in turns create cap-1 and cap-2 structure, respectively (Furuichi and Shatkin, 2000). Taken into consideration, it was shown that ribose 2^ʹ^-O-methylation of viral RNA cap provided a mechanism for viruses to overcome the host immune recognition (Daffis et al., 2010; Zust et al., 2011). Furthermore, it was recently reported that SARS-CoV nsp16 acts as 2ʹ-O-MTase which together with nsp10 give the rise to cap-1 structure (Bouvet et al., 2010). Interestingly, some of the honey bee compounds showed high affinity on 2ʹ-O-methylates. Among the FDA-approved medications, paritaprevir and teniposide influence the conversion of spike macromolecule, 2′-o-ribose methyltransferase, dihydroergotamine and venetoclax to nucleocapsid macromolecule and 2′-o-ribose methyltransferase. Among the natural products, amyrin (triterpenes), procyanidin, and proanthocyanidin (category of flavonoids) influence the activity of 2′-o-ribose methyltransferase (Kadioglu et al., 2020). It is noteworthy to state that the major compounds of propolis are triterpenoids that have a relative concentration of 74%, steroids, and diterpenoids (Elnakady et al., 2017). Raw propolis contains over three hundred different compounds that largely consist of triterpenes (50% w/w), waxes (25–30%), and phenolics (5–10%), volatile mono- and sesquiterpenes (8–12%) and the last compound gives propolis its typical pitchy odor (Huang et al., 2014).

### Potential Restrictive Properties Against Ribonucleic Acid-dependent RNA Enzyme

Ribonucleic acid-dependent RNA enzyme (RdRp) is considered a crucial enzyme for coronaviruses as it catalyzes the replication of ribonucleic acid from RNA templates. Importantly, a remarkable similarity in the sequences and cipher structures of RdRp were reported among the sequence of RdRp in severe acute respiratory syndrome coronavirus (SARS-CoV), SARS-CoV-2 and Middle East respiratory syndrome coronavirus (MERS-CoV) (Feng et al., 2020; Morse et al., 2020). Interestingly, Remdesivir (GS-5734) might represent a nucleoside ester analogue substance of RdRp since it showed broad-spectrum antiviral activity against many ribonucleic acid viruses, as well as filovirus, SARS-CoV and MERS-CoV(Tchesnokov et al., 2019; Gordon et al., 2020; Zhang and Liu, 2020). Furthermore, a recent report indicated that remdesivir improved the vital condition of a patient with COVID-19 that might represent a therapeutic target for SARS-CoV-2 (Holshue et al., 2020).

Interestingly, twelve completely different flavonoids were detected in propolis extracts namely, pinocembrin, acacetin, chrysin, rutin, luteolin, kaempferol, apigenin, myricetin, catechin, naringenin, galangin, and quercetin; 2 synthetic resin acids, caffeic acid and cinnamic acid (Volpi, 2004). Among others, myricetin has high binding affinity toward the RdRp of each SARS-CoV and SARS-CoV-2 with favorable materia medica properties. This compound has been consumed since a time long ago and does not possess any inherent toxicity besides exhibiting a broad range of therapeutic properties, suggesting the potential use of this natural compound as inhibitor for RdRp of SARS-CoV-2. However, additional *in vitro* and *in vivo* studies seem mandatory to validate its efficaciousness against SARS-CoV-2 (Singh et al., 2020).

## Potential Repressive Properties Against the Cytokine Storm

Revising the available literature, threatens of COVID-19 is partially related to cytokine storm which is defined as an exaggerated production of proinflammatory cytokines that results in multi organ system failure (Jose and Manuel, 2020; Tufan et al., 2020). It has been documented that COVID-19 infected patients with cytokine storm manifest high levels of cytokines, together with higher plasma levels of various interleukins (IL), including IL-2, IL-6, IL-7, IL-10, Granulocyte colony-stimulating factor (G-CSF), Interferon gamma (IFNγ), Microtubule-associated protein 1 alpha, and Tumor necrosis factor alpha (TNFα) (Costela-Ruiz et al., 2020; Mehta et al., 2020). Among others, IL6 is an inflammatory protein involved in cytokine storms that triggers the upregulation of T helper 1 (Th1) and T helper cell 2 (Th2) pathways (Kipar et al., 2006; Yan et al., 2018). In addition, whole bee venom down-regulates TNFα and IL-6 (Kim et al., 2011; Darwish et al., 2013; Shin et al., 2017). Moreover, bee venom is already utilized in some varieties of stylostixis for treatment of inflammatory arthritis (Lee et al., 2005). It was reported that cytokine IL10 down-regulates inflammatory cytokines like IL1 and TNF alpha in coronavirus infections (Cox, 1996). Bee venom (BV) was also reported to contain many enzymes, together with phospholipase A2 (PLA2), phospholipase B, spreading factor, acid enzyme and–glucosidase (Hossen et al., 2016). In a previous study, Park et al. (2015) showed that bee venom phospholipase A2 (BV PLA2) ameliorates allergic airway inflammation. This study found that BV PLA2 treatment causes diminished infiltration of neutrophils, eosinophils, lymphocytes, and macrophages in bronchoalveolar irrigation fluid (BLAF) (Park et al., 2015). Another study revealed that BV PLA2 includes a CD4^+^CD25 + Foxp3+ Treg cell-mediated protection against acute respiratory organ inflammation induced by actinotherapy (Shin et al., 2016). Interestingly, the exaggerated IL10 that was reported in beekeepers compared to the rest of population might be attributed to the chronic low level exposure to bee venom (Meiler et al., 2008). A recent study also proposed that beekeepers are often prevented from SARS-CoV-2 as results of this population developing a tolerance to bee stings (Yang et al., 2020). Clearly, this observation suggests that bee venom or product with active ingredients contained in bee venom can be used in people at high risk of serious COVID-19 to forestall or attenuate cytokine storm within the context of COVID-19.

## Limitations of the Studies Carried out in Honey Bee Compounds

Given the above information, natural products are among the therapeutic options for SARS-CoV-2 infection (Serkedjieva et al., 1992; Calixto, 2005; Maruta and He, 2020). Propolis is an example for honey bee compounds that can reduce and alleviate the symptoms of inflammatory diseases by affecting various metabolic cycles (Machado et al., 2012; Hori et al., 2013; Piñeros et al., 2020). However, found many restrictions were found for the approval and acceptance of these substances as a health-promoting supplement in various countries since these compounds e.g., propolis products are not standardized and vary in their components and biological activity among countries and even at a regional level, and therefore, faced many relevant criticism (Bankova, 2005; Toreti et al., 2013; Miguel et al., 2014). However, it should be stressed that standardized propolis products e.g., standardized Brazilian propolis extract blend have recently become available to overcome this major drawback and showed higher safety profile and major effectiveness for treatment of many pathological conditions (Berretta, 2007; Berretta et al., 2012; Berretta et al., 2017; Silveira et al., 2019; Zaccaria et al., 2019). Therefore, standardized propolis is considered an example for natural products that can be used a nutraceutical or functional food resource that might provide a promising safe and easy to administer therapeutic for fighting COVID-19 pandemic (Fielding et al., 2020).

## Conclusions, Limitations, and Future Perspectives

Given the above information, the current pandemic status of COVID-19 calls the urgent need to develop non-traditional novel drug targets and vaccines for combating the disease. Interestingly, several previous studies revealed the physiological and therapeutic actions of bee products (propolis, bee pollen, bee venom and honey) and their components, implicating their potential role in controlling various pathological conditions including COVID-19. However, it should be borne in mind that studies on bee bioactive compounds and their role in COVID-19 are limited and bee products may have different compositions. Taken together, the current review suggests further future studies on exploring the potential beneficial use of bee products besides investigation their detailed chemical analysis. This detailed information might provide clues for their use as potential drug targets for combating CoVID-19 either alone or in association with other drugs.
